# Plant metabolites targeting the AMPK pathway: advances in the mechanisms of action in the context of MAFLD

**DOI:** 10.3389/fphar.2026.1873469

**Published:** 2026-07-16

**Authors:** Meiyan Li, Han Wang, Dandan Cui, Min Li, Yuting Niu, Jingyin Yu, Xiaolin Dang, Jue Wang

**Affiliations:** 1 College of Pharmacy, Shaanxi University of International Trade & Commerce, Xi’an, China; 2 School of Pharmacy, Chengdu University of Traditional Chinese Medicine, Chengdu, China

**Keywords:** MAFLD, AMPK signaling pathway, plant metabolites, mechanism of action, clinical translation

## Abstract

Metabolic dysfunction-associated fatty liver disease (MAFLD) is recognized as the hepatic manifestation of metabolic syndrome. Hepatic steatosis resulting from impaired energy metabolism constitutes the core of its pathogenesis, and this disease has become a global public health concern. AMP-activated protein kinase (AMPK) is widely expressed in high-energy-consuming organs and functions as a vital energy sensor that maintains systemic energy homeostasis. Current preclinical *in vitro* and *in vivo* evidence demonstrates that AMPK activation modulates multiple MAFLD-related pathological processes, including enhancing cellular autophagy, regulating lipid metabolism, reducing inflammatory and oxidative damage, improving IR, and mitigating mitochondrial dysfunction. Plant metabolites have attracted increasing research attention for targeting the AMPK pathway in MAFLD basic research, due to their multi-target regulatory characteristics and low adverse reaction profiles. Accordingly, based on the intrinsic connection between the AMPK signaling pathway and MAFLD, this review summarizes the research progress regarding the pharmacological mechanisms of plant metabolites acting on the AMPK pathway in MAFLD intervention. Notably, most studies included in this review are preclinical experiments conducted *in vitro* and *in vivo*, with scarce supporting clinical data. Further efforts are still needed to advance the translation of these findings and confirm their clinical potential. This review systematically summarizes recent basic research progress and identifies unresolved issues, so as to offer a theoretical basis for subsequent mechanistic studies and translational exploration of traditional Chinese medicine against MAFLD.

## 1 Introduction

Metabolic dysfunction-associated fatty liver disease (MAFLD), a major chronic metabolic disease with high global prevalence, presents predominantly as hepatic steatosis. In 1986, Shaffner and Thaler first introduced the term non-alcoholic fatty liver disease (NAFLD). In 2020, an international expert panel revised its diagnostic framework by abandoning the original exclusion-based diagnostic paradigm of NAFLD and recommended its reclassification as MAFLD, whereby metabolic dysfunction and relevant risk factors were integrated into core diagnostic criteria. In June 2023, multiple leading international hepatology societies further proposed replacing MAFLD with metabolic dysfunction-associated steatotic liver disease (MASLD) following a multisociety Delphi consensus. Collectively, these three terminologies describe an identical hepatic disease, distinguished solely by sequential refinements in diagnostic criteria. Considering the broad publication timeframe of cited references and the limited availability of comprehensive investigations focused on the newly introduced MASLD, MAFLD is consistently employed as the standard terminology across the present review (Beygi et al., 2024). Despite these nomenclature updates, MAFLD remains a highly prevalent liver disease with a global prevalence of 32.4%, representing a growing public health burden (Liu Y. et al., 2025; Peng et al., 2024). MAFLD has a complex pathogenesis. Lipid metabolism disorders lead to excessive fat deposition in the liver, which drives disease progression. The condition may progress to metabolic-associated steatohepatitis, cirrhosis, liver failure, and eventually advanced hepatocellular carcinoma, resulting in sharply elevated mortality (He et al., 2025; Qiao et al., 2024).

AMP-activated protein kinase (AMPK) is a protein kinase consisting of a heterotrimeric complex containing α, β, and γ subunits. The α-subunit serves as the core catalytic subunit, and its Thr172 site can be phosphorylated by upstream kinases to activate multiple AMPK-mediated cascade reactions. The β-subunit is mainly involved in glycogen synthesis and metabolism. The γ-subunit functions as a sensor for changes in the AMP/ADP ratio. It has been demonstrated that AMP is a physiological regulator of AMPK activation (Gowans et al., 2013). AMP binds to the Bateman domain of the γ subunit, enhancing the phosphorylation of Thr172 on the α subunit by LKB1 or CaMKKβ through an allosteric effect, thereby sustaining the persistent activation of AMPK (Oakhill et al., 2011).

The development of MAFLD involves the “multiple hits” mechanism, and disordered energy metabolism-driven hepatic steatosis is recognized as its core pathological feature. AMPK is abundantly expressed in high-energy metabolic organs, including the liver, skeletal muscle, and adipose tissue, and acts as a crucial energy sensor governing systemic energy homeostasis. AMPK modulates various pathological changes such as autophagy, lipid metabolism, insulin resistance (IR), inflammatory responses, oxidative stress, and mitochondrial dysfunction, making it a highly promising therapeutic target for MAFLD. Plant metabolites, well recognized for their multi-target effects and favorable safety profiles, exert therapeutic effects on MAFLD by targeting the AMPK signaling pathway. While previous reviews have summarized the interplay between AMPK and plant metabolites in MAFLD research, most publications lack in-depth discussion on the structural classification of plant metabolites, as well as the barriers to clinical translation and related safety concerns. This review adopted “NAFLD/MAFLD/MASLD” and “AMPK signaling pathway” as the core retrieval keywords, combined with supplementary search terms related to plant metabolites. Relevant studies published in the PubMed and CNKI databases over the past decade were systematically retrieved. All research was processed through a standardized stepwise screening procedure, including initial database searching, duplicate elimination, title and abstract screening, and full-text rescreening. Ultimately, only high-quality original pharmacological studies with clearly defined experimental models, complete parameters of intervention dosage and duration, and detailed pharmacological mechanism data were included in this review. It systematically elaborates the pharmacological mechanisms of plant metabolites targeting AMPK, and further classifies the existing metabolites according to their chemical structures. Importantly, this review systematically addresses these gaps and proposes future research directions. This review aims to provide a systematic reference for the in-depth research and clinical translation of AMPK-targeted plant metabolites in the prevention and treatment of MAFLD.

## 2 Mechanism of action of the AMPK signaling pathway in MAFLD

**FIGURE 1:**
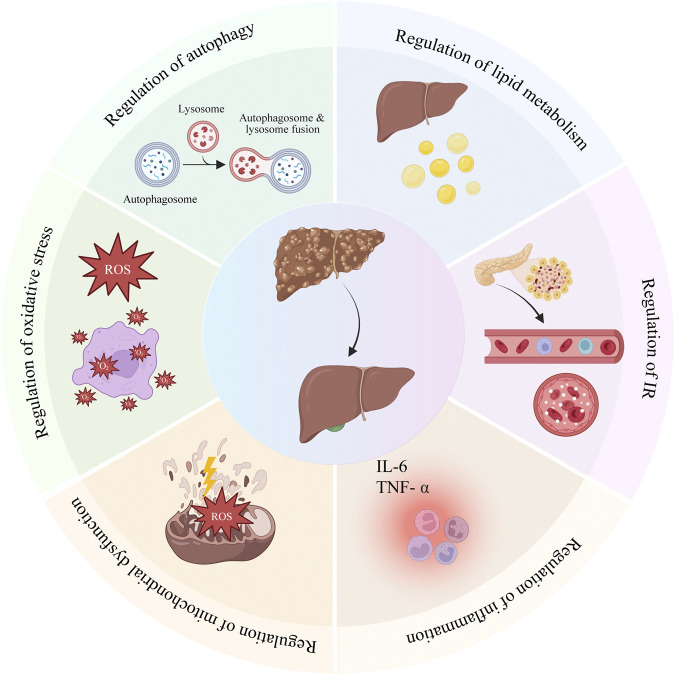
Six action pathways by which targeted AMPK intervention alleviates MAFLD.

As an energy balance sensor, AMPK exerts its ameliorative effects on MAFLD through multiple mechanisms, including regulating lipid metabolism, inhibiting IR and inflammatory responses, alleviating mitochondrial dysfunction and oxidative stress, and inducing autophagy. These multifaceted biological functions identify AMPK as an essential potential therapeutic target for MAFLD. Details are shown in Figure 1.

### 2.1 Regulation of lipid metabolism

It is widely recognized that the liver is the key organ for lipid oxidation, synthesis, and metabolism. Elevated hepatic fatty acid synthesis is coupled with suppressed fatty acid catabolism. In other words, an imbalance between influx and efflux of free fatty acids (FFAs) triggers hepatic lipid metabolic dysfunction, a pivotal event driving MAFLD progression (Shen et al., 2024; Sun et al., 2025). AMPK primarily regulates lipid metabolism by reducing lipid synthesis and promoting fatty acid oxidation. Sirtuin 1 (SIRT1) is an essential regulator of energy homeostasis (Yang et al., 2023). It has been demonstrated that AMPK can inhibit transcription factors related to lipid synthesis by regulating SIRT1, with sterol regulatory element-binding protein-1c (SREBP-1c) serving as a key transcription factor regulated by AMPK. SREBP-1c is a master transcriptional controller of lipid biosynthesis. It translocates into the nucleus, binds to sterol regulatory elements (SREs), and initiates transcriptional expression of downstream target genes *FAS* and *SCD1*, thereby accelerating fatty acid generation and triglyceride (TG) synthesis (Li et al., 2016; Nguyen et al., 2021). Therefore, activating AMPK effectively alleviates MAFLD by negatively regulating SREBP-1c, thereby reducing its activation, cleavage, and nuclear translocation, suppressing lipogenic factors including FAS, acetyl-CoA carboxylase 1 (ACC1), and SCD1, and decreasing lipid synthesis and deposition (Chen et al., 2019; Li et al., 2011; Li, 2024). AMPK accelerates fatty acid oxidation by regulating SIRT1 and activating the PPARα/CPT-1 pathway (Zhang J. et al., 2023; Zhang N. et al., 2023). As a master transcriptional regulator, PPARα governs mitochondrial fatty acid β-oxidation and cytosolic FFA transport. Under the regulation of PPARα and its downstream effectors CPT1 and CPT2, cytosolic fatty acyl-CoA is transported into mitochondria and catabolized via fatty acid oxidation to yield acetyl-CoA. Meanwhile, cytosolic acetyl-CoA functions as the primary precursor for *de novo* lipogenesis (DNL). Accumulating evidence indicates that AMPK activation facilitates nuclear translocation of PPARα and upregulates its target gene *CPT1A*, consequently accelerating hepatic fatty acid β-oxidation (Liu X. et al., 2025).

**FIGURE 2:**
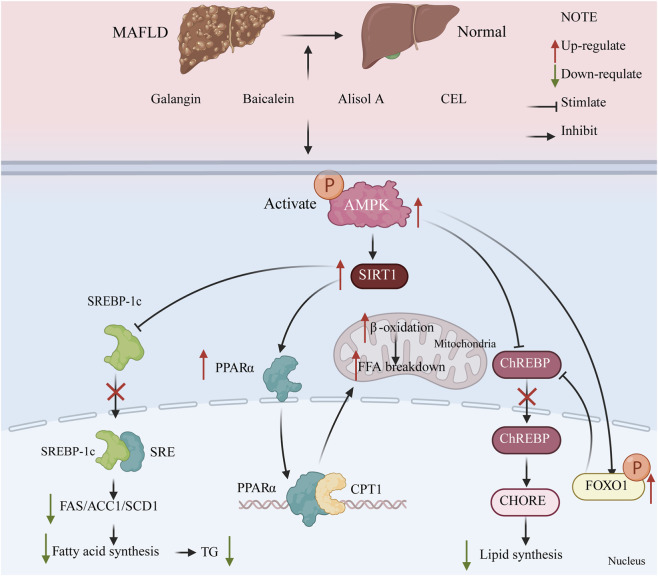
The mechanism of action of improving MAFLD by targeting AMPK to regulate lipid metabolism.

Beyond SREBP-1c-dependent lipogenesis and PPARα-governed fatty acid oxidation, DNL represents another vital driver of hepatic lipid accumulation during MAFLD development, which is tightly controlled by another core transcriptional factor ChREBP. Elevated DNL further promotes hepatic lipid deposition. SREBP1c and ChREBP synergistically modulate hepatic glycolysis and lipogenic gene expression, acting as core lipogenic transcription factors for DNL. ChREBP-regulated hepatic fructose metabolism is tightly correlated with MAFLD progression and contributes to DNL-mediated hepatic steatosis (Régnier M et al., 2023). A previous study demonstrates that temozolomide alleviates MAFLD by suppressing ChREBP-mediated DNL by activating the AMPK/FOXO1 pathway (Zhang et al., 2020). The detailed mechanisms by which plant metabolites intervene in MAFLD by regulating lipid metabolism are illustrated in Figure 2.

### 2.2 Regulation of IR

Insulin suppresses hepatic gluconeogenesis. Under normal insulin sensitivity, hepatocytes take up circulating glucose and synthesize hepatic glycogen, thereby stabilizing blood glucose levels. IR is a pathological state characterized by reduced insulin sensitivity or diminished cellular responsiveness to insulin. IR causes decreased insulin responsiveness in the liver, adipose tissue, and other metabolic organs, which increases lipolysis, leading to excessive FFAs influx into the liver and hepatic lipid accumulation. To compensate for elevated blood glucose and glucagon levels, pancreatic β-cells secrete excess insulin, resulting in hyperinsulinemia and further exacerbation of IR (Zhang and Feng, 2022). Concurrently, IR selectively impairs the glucose-lowering effect of insulin, thereby enabling hepatic DNL to promote lipogenesis via SREBP1-c activation (Huang, 2022).

The AMPK pathway is critically involved in the development of IR. AMPK functions as a downstream effector kinase of LKB1. Enhanced LKB1 expression attenuates hypothalamic inflammation, elevates insulin sensitivity, and facilitates glucose uptake and metabolism, ultimately improving IR. Subsequently, AMPK promotes fatty acid oxidation and glucose uptake, while inhibiting lipogenesis and cholesterol synthesis, thereby alleviating IR. The inflammatory cytokines further impair insulin signaling, exacerbating IR. AMPK activation suppresses inflammatory signaling pathways such as NF-κB, thereby reducing inflammatory responses and ameliorating IR. Activation of AMPK also promotes Nrf2 nuclear translocation and binding to ARE. This binding event triggers the expression of multiple antioxidant factors, which protect cells from oxidative stress damage and reduce ROS levels to improve IR (Yan et al., 2018). Furthermore, AMPK modulates adipocyte function to alleviate IR. Adipocytes produce and secrete various adipokines, including lipocalin and leptin, which participate in systemic regulation of insulin sensitivity. Notably, AMPK activation in peripheral adipose tissue can modulate the PKA pathway and induce adipocyte browning, which subsequently enhances systemic insulin sensitivity and decreases hepatic FFA delivery. These effects work through cross-organ coordination between adipose tissue and the liver, contributing to suppression of MAFLD progression (Ge and Zhang, 2024).

**FIGURE 3:**
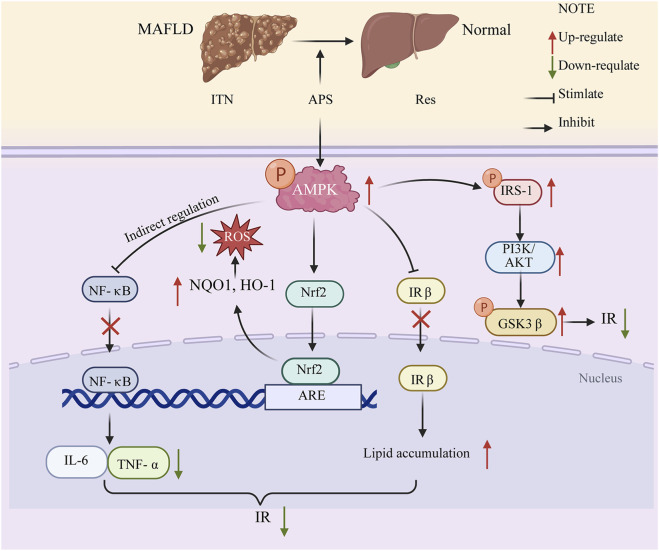
The mechanism of action of improving MAFLD by targeting AMPK regulation of IR.

Consistent with the above regulatory network, multiple plant metabolites exert anti-MAFLD effects by targeting AMPK to reverse hepatic IR. The saturated fatty acid (PA) is a common FFA that triggers IR via inducing lipid accumulation in hepatocytes. Treatment with the mangiferin derivative X-4 dramatically ameliorated PA-induced IR and lipid accumulation, while suppressing cellular insulin receptor β (IRβ) nuclear translocation. IRβ is essential for insulin signal transduction. The experiments further revealed that the inhibitory effect of X-4 on IRβ nuclear translocation was markedly weakened when AMPK was blocked by the inhibitor Compound C. These results demonstrate that activation of the AMPK/IRβ signaling pathway contributes significantly to the reduction of hepatic lipid accumulation (Fan, 2023). As another representative plant metabolite, Astragalus polysaccharides (APS) act on AMPK as their primary upstream target, restoring insulin signaling and improving hepatic IR by regulating the IRS-1-PI3K-AKT-GSK3β signaling pathway (Sun et al., 2019). Collectively, plant metabolites alleviate IR through activation of the AMPK pathway, thereby ameliorating MAFLD. The detailed mechanisms by which plant metabolites ameliorate MAFLD via inhibiting IR are illustrated in Figure 3.

### 2.3 Regulation of inflammation

**FIGURE 4:**
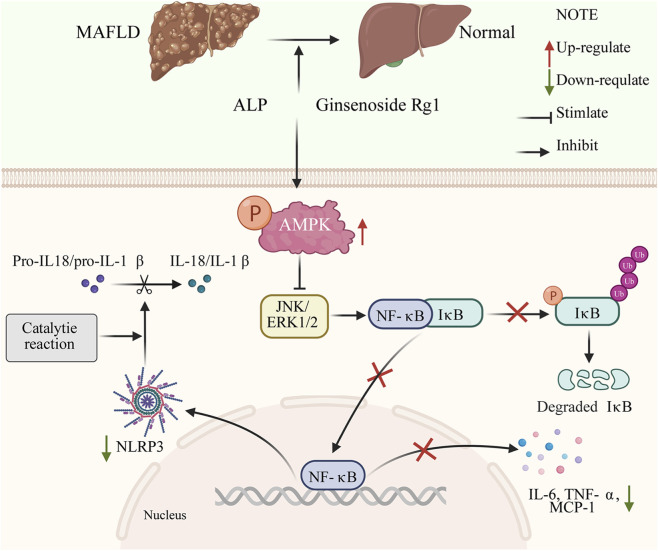
The mechanism of action of improving MAFLD by targeting AMPK to regulate inflammation.

Large amounts of FFAs accumulate in the liver tissues of patients with MAFLD. Overaccumulation of lipids leads to intracellular ROS overproduction, thereby inducing an inflammatory response. Targeted AMPK activation can mitigate these inflammatory responses. Pro-inflammatory cytokine production is primarily regulated by the NLRP3 inflammasome (Deng et al., 2025). The NF-κB pathway plays a vital regulatory role in inflammatory responses. NF-κB activation upregulates the expression and assembly of the NLRP3 inflammasome, which subsequently cleaves pro-IL-1β and pro-IL-18 into their biologically active mature forms (Jing et al., 2022). Inhibition of NF-κB nuclear translocation efficiently suppresses NLRP3 inflammasome activation and alleviates inflammatory injury in MAFLD. Dong et al. found that lipocalin could target the AMPK protein, modulating its downstream JNK/Erk1/2 signaling pathway, inhibiting NF-κB activation, and further suppressing PA-induced activation of the NLRP3 inflammasome in hepatocytes (Dong et al., 2020). Previous mechanistic studies have confirmed that Compound C (a selective AMPK inhibitor) blocks NF-κB nuclear translocation in PA-stimulated HepG2 cells and abolishes the hepatoprotective effects of PDS-C, a ginsenoside metabolite. These findings validate that AMPK modulates the NF-κB pathway, which in turn regulates the expression of several pro-inflammatory cytokines such as TNF-α and IL-6 (Mi et al., 2024). The detailed mechanisms by which plant metabolites alleviate MAFLD via suppression of inflammatory responses are illustrated in Figure 4.

### 2.4 Regulation of mitochondrial dysfunction

Mitochondria are critical organelles for intracellular energy production and a major cellular site for adenosine triphosphate (ATP) generation and lipid oxidation (Lin et al., 2021). Dysfunctional mitochondria may impair intracellular lipid oxidation, causing excessive lipid accumulation in the cytoplasm. It is well established that intact mitochondrial function is indispensable for restraining MAFLD progression.

**FIGURE 5:**
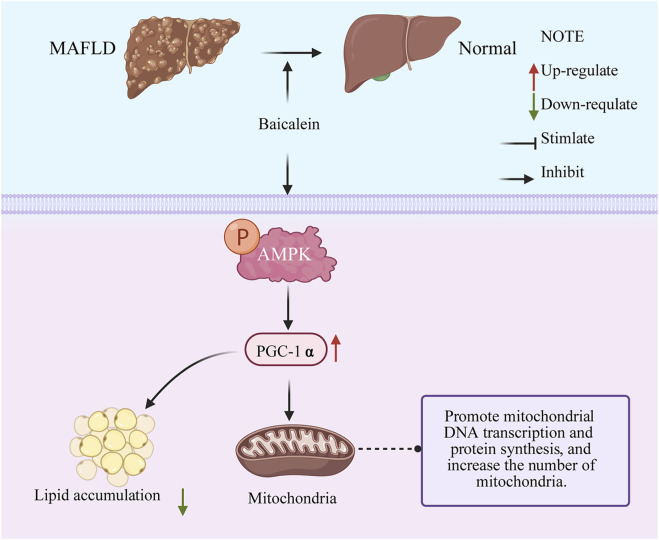
The mechanism of action of improving MAFLD by targeting AMPK to regulate mitochondrial dysfunction.

Mitochondrial dysfunction or reduced mitochondrial content leads to excessive ROS production, which contributes to the pathogenesis of chronic diseases such as MAFLD and diabetes. PGC-1α is a major regulator of mitochondrial biogenesis. By interacting with multiple nuclear receptors and transcription factors, PGC-1α promotes mitochondrial DNA transcription and protein synthesis, thereby increasing mitochondrial content and improving mitochondrial function (Yuan et al., 2023). During MAFLD progression, downregulation of PGC-1α expression results in impaired mitochondrial oxidative capacity in the liver and triggers hepatic steatosis. Phosphorylated AMPK (p-AMPK) alleviates lipid accumulation, enhances mitochondrial function, and upregulates antioxidant protein expression by regulating PGC-1α, thereby reducing ROS production (Cheng et al., 2024; Li et al., 2022). The detailed mechanisms by which plant metabolites alleviate MAFLD via regulation of mitochondrial dysfunction are illustrated in Figure 5.

### 2.5 Regulation of oxidative stress

Oxidative stress is closely related to lipogenesis and lipolysis. Excessive hepatic lipid accumulation in MAFLD triggers mitochondrial dysfunction, which generates large quantities of FFAs. FFAs build up within hepatocytes and drive abnormally elevated ROS levels; subsequent oxidative stress induces cellular injury and promotes hepatocyte apoptosis, thereby accelerating MAFLD progression (Yang et al., 2024). Oxidative stress refers to a pathological state characterized by an imbalance between oxidative and antioxidant systems. This imbalance leads to excessive ROS accumulation and lipid peroxidation, ultimately causing cellular, tissue, and organ damage.

Nrf2 is an endogenous redox-sensitive transcription factor that can participate in antioxidant defense by modulating inflammation and oxidative stress (Zhang et al., 2025). Nrf2 is also an important downstream target of AMPK. Under physiological conditions, Nrf2 binds to Keap1 and is sequestered in the cytoplasm. Keap1 contains abundant reactive cysteine residues and acts as a cellular oxidative stress sensor. Upon oxidative modification of Keap1 cysteine residues, Keap1 loses the capacity to bind Nrf2, leading to its stabilization. Nrf2 then translocates into the nucleus and binds to AREs to upregulate downstream antioxidant enzymes, such as HO-1, NQO1, SOD, and GSH-Px, which in turn ameliorates MAFLD-related hepatic damage (Biao et al., 2023).

**FIGURE 6:**
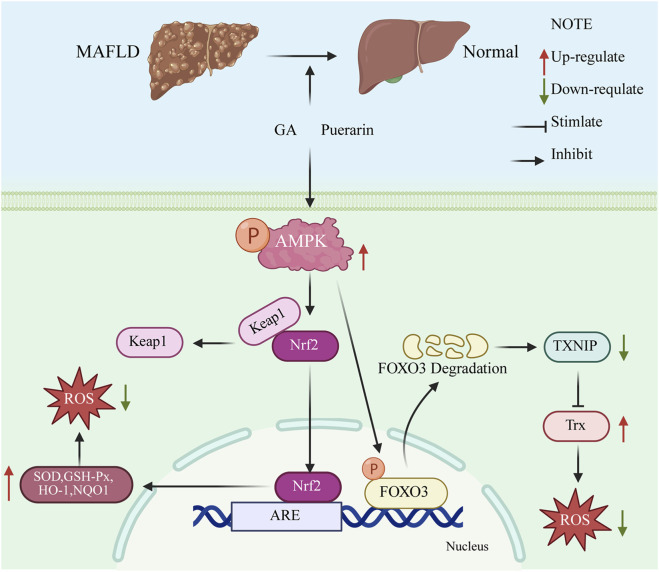
The mechanism of action of improving MAFLD by targeting AMPK to regulate oxidative stress damage.

TXNIP, a critical redox regulator, exacerbates intracellular oxidative stress by inhibiting thioredoxin activity and weakening cellular antioxidant defenses. It has been established that AMPK counteracts oxidative stress by downregulating FOXO3 and TXNIP expression (Huang et al., 2022). The detailed mechanisms by which plant metabolites ameliorate MAFLD via alleviation of oxidative stress are illustrated in Figure 6.

### 2.6 Regulation of autophagy

Autophagy is a pathway that degrades proteins, impaired organelles, and various macromolecules via the autophagy-lysosomal system. Autophagy plays a critical role in regulating hepatic lipid metabolism. mTOR is a primary target of the mammalian autophagy pathway and exists in two distinct complexes, mTORC1 and mTORC2. mTORC1 phosphorylates ULK1 at the serine 757 site (Ser757), inhibiting its activity and suppressing autophagy. AMPK activation promotes autophagy by phosphorylating tuberous sclerosis 2 (TSC2) and the Raptor protein in mTORC1, thereby inhibiting mTORC1 activity and upregulating ULK1 activity. Moreover, AMPK can phosphorylate ULK1 at Ser555, Ser317, and Ser777, which directly activates ULK1, promotes autophagy in hepatocytes, and facilitates lipid droplet catabolism. These findings indicate that the AMPK/mTOR/ULK1 signaling axis is one of the important pathways to regulate autophagy (Biao et al., 2024a; Biao et al., 2024b). mTOR is also a nutrient-sensitive kinase. Under nutrient-rich conditions, mTOR can promote lipid synthesis while inhibiting catabolism. In contrast, AMPK can inhibit mTOR activity to modulate lipid metabolism. The conversion of LC3-I to LC3-II occurs during the formation of autophagosomes. Hence, elevated LC3-II levels correlate positively with autophagy activity. Liao et al. demonstrated that in an OA-induced MAFLD cellular model, p-AMPK/AMPK and LC3-II/I protein levels were downregulated, while p-mTOR/mTOR and p62 levels were upregulated, and pioglitazone reversed these alterations. In addition, pioglitazone treatment downregulated SREBP-1 expression and reduced lipid accumulation in the MAFLD cell model (Liao et al., 2022).

**FIGURE 7:**
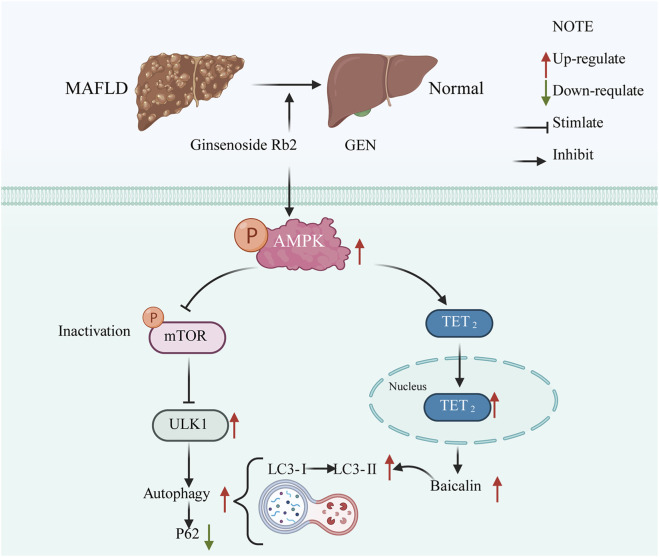
The mechanism of action of improving MAFLD by targeting AMPK to regulate autophagy.

*TET2* mediates DNA demethylation and was originally identified as a tumor suppressor gene in acute myeloid leukemia. Disruption of the DNA methylation–demethylation equilibrium is critically implicated in the pathogenesis of metabolic diseases (Feinberg et al., 2010). Accumulating evidence reveals that AMPK activation upregulates TET2 expression, which subsequently increases autophagy-related protein levels, reduces lipid deposition, and alleviates MAFLD (Li et al., 2021). The detailed mechanisms by which plant metabolites alleviate MAFLD via induction of autophagy are illustrated in Figure 7.

## 3 Targeting AMPK signaling pathway against MAFLD by plant metabolites

### 3.1 Saponins

*Panax ginseng* C. A. Mey (Araliaceae) was first recorded in Shennong Ben Cao Jing, with the effects of tonifying qi, strengthening the spleen, tranquilizing the mind, and improving intelligence (Zhou et al., 2024). Ginsenosides are the primary metabolites of ginseng, among which ginsenosides Rb2, Rg1, and Rg5 are the most extensively investigated (Kim, 2018). Huang et al. observed that ginsenoside Rb2 reduces lipid and blood glucose levels in mice. Both *in vitro* and *in vivo* studies have demonstrated that ginsenoside Rb2 upregulates the protein levels of p-AMPK/AMPK and LC3-II, while downregulating p-mTOR/mTOR, p-ULK1, and p62 expression. These findings indicate that ginsenoside Rb2 ameliorates metabolic disorders by activating the AMPK pathway and further enhancing hepatic autophagy (Huang et al., 2017). Xiao et al. established a PA-induced lipid accumulation model in HepG2 cells and discovered that ginsenoside Rg1 effectively ameliorated PA-induced lipid accumulation. Ginsenoside Rg1 upregulated p-AMPK levels, leading to ACC inactivation and suppression of SREBP-1c and FAS expression. Further treatment of AMPK inhibitors significantly attenuated the protective effects of Rg1 on cells (Xiao et al., 2019a). In subsequent investigations, Xiao et al. observed that ginsenoside Rg1 also diminished levels of inflammatory cytokines, reduced degradation of IκBα, and blocked nuclear translocation of the p65 subunit (Xiao et al., 2019b). Shi et al. established an MAFLD model by feeding a high-fat diet (HFD) to mice. Compared with normal mice, HFD-fed mice exhibited significantly elevated blood glucose levels, which were effectively reversed by ginsenoside Rg5 treatment. HE and Oil Red O staining results demonstrated that ginsenoside Rg5 markedly ameliorated HFD-triggered hepatic steatosis, inflammatory infiltration, and lipid accumulation. Meanwhile, ginsenoside Rg5 significantly restored oxidative stress-related indicators in MAFLD mice. Western blot analysis verified that the model group presented downregulated expression of LKB1, p-AMPK, and PPARγ, as well as upregulated levels of p-mTOR and p-NF-κB, whereas ginsenoside Rg5 reversed these alterations. Mechanistically, ginsenoside Rg5 activates upstream LKB1, thereby initiating the LKB1/AMPKα/PPARγ signaling pathway. Activated AMPK further negatively modulates the downstream mTOR and NF-κB pathways. This dual regulatory effect promotes hepatic lipolysis, enhances autophagy, and suppresses inflammatory responses, ultimately mitigating hepatic lipid accumulation and improving HFD-induced MAFLD (Shi, 2024).

### 3.2 Flavonoids

Naringenin is the principal metabolite derived from *Citrus × aurantium* L. f. *aurantium* (Rutaceae). Relevant animal experiments have verified its ability to ameliorate MAFLD induced by a high-fat diet in rats. Studies using oleic acid-stimulated L02 hepatocyte models revealed that Compound C, a specific AMPK inhibitor, markedly reversed the lipid-lowering and autophagy-regulating effects of naringenin. Molecular docking analysis confirmed that naringenin forms hydrogen bonds with the amino acid residues Ala205, Ala227, Ser227, and Asp317 on the AMPKγ1 subunit, which are the endogenous AMP-binding sites. Mechanistic experiments in 3T3-L1 adipocytes demonstrated that naringenin activates the CaMKKβ/AMPK/ACC signaling pathway and inhibits aberrant cellular autophagy. In conclusion, naringenin exerts its therapeutic effects against MAFLD through two pathways: indirect activation of AMPK *via* upregulation of CaMKKβ, and direct binding to the AMPKγ1 subunit (Yang et al., 2021). Atractylenolide III (ATL III) is the primary metabolite isolated from *Atractylodes macrocephala* Koidz (Asteraceae), and has been reported to exert antitumor, antioxidant, and antibacterial activities. Li et al. reported that ATL III treatment attenuated lipid accumulation, restored AdipoR1 expression, decreased ROS and MDA levels, and enhanced the activities of GSH-Px and SOD. Mechanistically, LKB1 acts as an indispensable upstream kinase of AMPK and serves as a critical intermediate downstream of AdipoR1 to phosphorylate and activate AMPK, thereby initiating the subsequent signaling cascade. Further mechanistic studies have revealed that ATL III activates the LKB1/AMPK and SIRT1 pathways, thereby promoting the expression of downstream target molecules, including Nrf2 and PGC1α. Importantly, the suppressive effect of ATL III on FFA-induced lipid droplet formation in cells was abolished by pretreatment with AMPK or SIRT1 inhibitors. The present study verified that ATL III exerts therapeutic effects against MAFLD by activating the AdipoR1-mediated LKB1/AMPK-SIRT1 pathway, thereby alleviating lipid accumulation and oxidative stress (Li et al., 2022; Zheng et al., 2024). *Erigeron breviscapus* (Vaniot) Hand.-Mazz (Asteraceae) is primarily distributed in Yunnan Province, and its major metabolite is scutellarin (SCU). Zou established an animal model of MAFLD, which revealed that SCU treatment dramatically reduced body weight, liver weight, blood glucose levels, lipid aggregation, and hepatic fibrosis in rats. Moreover, Western blot analysis indicated that SCU effectively upregulated the expression of SIRT3, P-AMPK, and P-ACC proteins. As a crucial upstream regulatory molecule of AMPK, SIRT3 activation further promoted the phosphorylation of AMPK and ACC, thereby mediating the lipid-regulating effects of SCU. A lipid metabolism disorder model was constructed in LO2 cells using PA, and the *in vitro* results were highly consistent with the *in vivo* findings. It has been confirmed that SCU alleviates the progression of MAFLD by activating the SIRT3/AMPK/ACC signaling pathway (Zou, 2024). Galangin is a natural flavonoid derived from the roots of *Alpinia officinarum* Hance (Zingiberaceae). In a study by Cen et al., galangin markedly alleviated lipid droplet deposition induced by either a high-fat diet or oleic acid stimulation. Collagen-I and α-SMA serve as canonical biomarkers of hepatic fibrosis. RT-qPCR and Western blot analyses further demonstrated that galangin dose-dependently downregulated the mRNA expression of lipogenic genes (SREBP-1c, FAS, and ACC) and reduced the protein levels of fibrotic markers (Collagen-I, α-SMA), while increasing AMPK phosphorylation. Collectively, these findings indicate that galangin improves hepatic lipid accumulation and liver fibrosis in model rats, probably through the activation of AMPK signaling pathway (Cen et al., 2022). Zhang’s study demonstrated that baicalein, the metabolite derived from *Scutellaria baicalensis* Georgi (Lamiaceae), decreased fructose-induced elevation of nuclear n-SREBP1c and ChREBP protein levels in rat hepatocytes. It also downregulated ACC expression and upregulated the expression of CPT1α, p-AMPK, and PGC1α. These findings indicated that baicalein suppressed MAFLD by decreasing ACC and SREBP1c activity, thereby reducing hepatic DNL and promoting fatty acid oxidation through AMPK signaling (Zhang N. et al., 2023; Zhang, 2023). Puerarin is an isoflavone metabolite derived from *Pueraria montana* var. *lobata* (Willd.) Maesen & S.M.Almeida ex Sanj. & Pred. (Fabaceae) (Kang et al., 2015). Han et al. established a mouse MAFLD model by feeding mice a high-fat diet for 12 consecutive weeks. The study confirmed that puerarin exerts therapeutic effects on MAFLD by regulating the AMPK/Nrf2/NF-κB signaling pathway, alleviating hepatic lipid deposition, liver injury, oxidative stress, and inflammatory responses (Han et al., 2026).

### 3.3 Polysaccharides

Zeng et al. discovered that *Arctium lappa* L (Asteraceae) polysaccharide (ALP) effectively reduced lipid droplet accumulation in HepG2 cells. Compared with OA-induced cells, the contents of TG, AST, and ALT were significantly decreased following ALP intervention. ALP upregulated AMPK and CPT1 while downregulating ACC1 mRNA expression. TLR4 serves a critical function in regulating glycolipid metabolism. In glucose metabolism, specific TLR4 knockout improves glucose tolerance and IR. In lipid metabolism, TLR4 modulates the expression of lipogenic genes, suppresses preadipocyte differentiation, and lowers lipid accumulation. Zeng et al. further explored the anti-inflammatory potential of ALP. Their findings revealed that ALP substantially downregulated both NF-κB and TLR4 expression. These results suggested that ALP exerts a beneficial regulatory effect on glycolipid metabolism in HepG2 cells, potentially mediated by the AMPK/TLR4/NF-κB pathway (Zeng, 2023; Zeng et al., 2023). Sun et al. induced IR in mice by administering an HFD for 8 weeks. Astragalus polysaccharide (APS), a polysaccharide metabolite derived from *Astragalus mongholicus* Bunge (Fabaceae), dramatically ameliorated HFD-induced IR and hepatic lipid accumulation. It enhanced IRS-1, AMPK, and ACC phosphorylation, and upregulated the expression of ATG3 as well as the ratio of LC3-II/LC3-I. These findings indicate that APS treatment attenuates lipid accumulation and restores autophagy by activating AMPK (Sun et al., 2019). Yu et al. reported that pachymaran, a polysaccharide metabolite derived from *Poria cocos* (Schw.) Wolf (Polyporaceae) downregulated HFD-induced elevations in TC and TG levels. HE staining indicated that HFD triggered hepatic steatosis as well as lymphocyte and macrophage infiltration in mice, which was effectively ameliorated by the polysaccharide. Western blot analysis revealed that the expression levels of CaMKKβ and p-AMPK rose in a dose-dependent manner with increasing Pachymaran concentration. These findings suggest that the underlying pathway by which Pachymaran ameliorates MAFLD may involve regulating the AMPK pathway and alleviating lipid metabolic disorders (Yu et al., 2025).

### 3.4 Terpenoids

Paeoniflorin is a monoterpene glucoside derived from *Paeonia lactiflora* Pall (Paeoniaceae). Li et al. confirmed that paeoniflorin alleviated IR and hepatic steatosis in rats induced by 20% fructose. Mechanistic studies revealed that paeoniflorin regulated the AMPK/SREBP-1c signaling pathway, downregulated the mRNA expression of downstream targets SCD-1 and FAS, and modulated the phosphorylation level of ACC1, thereby exerting therapeutic effects. Given that both LKB1 and CaMKKβ can mediate AMPK activation, the authors further explored the action mode of paeoniflorin on AMPK. The results demonstrated that paeoniflorin markedly upregulated LKB1 protein expression, while having no obvious effect on CaMKKβ. Collectively, these findings suggest that paeoniflorin activates the LKB1/AMPK signaling axis to promote fatty acid β-oxidation and inhibit lipogenesis, ultimately exerting a hepatoprotective effect (Li et al., 2018). Alisol A is a triterpenoid metabolite isolated from *Alisma plantago-aquatica* subsp. orientale (Sam.) Sam (Alismataceae). Ho et al. established a metabolic injury model by feeding mice a high-fat diet. They found that Alisol A reversed obesity-related elevations in TC, TG, LDL-C, FFAs, and FABP4. Oil red O staining confirmed that Alisol A decreased lipid accumulation. Glucose metabolism was assessed via IP-GTT and IP-ITT, showing that Alisol A remarkably ameliorated glucose tolerance and insulin sensitivity in mice. Further analysis of genes involved in fatty acid synthesis and β-oxidation revealed that Alisol A upregulated the mRNA expression of CAT, CPT2, and ECHS1. Western blot analysis showed that Alisol A restored the suppressed expression of p-AMPK and p-ACC induced by a high-fat diet, while simultaneously reducing the elevated expression of SREBP-1c. In summary, these findings suggest that Alisol A ameliorates metabolic disorders through activation of the AMPK pathway (Ho et al., 2019). Huang et al. discovered that triptolide, a diterpenoid metabolite from *Tripterygium wilfordii* Hook.f. (Celastraceae), plays a significant role in ameliorating MAFLD by boosting the phosphorylation of AMPK and ACC1, reducing inflammatory mediators, and enhancing fatty acid oxidation in animal experiments (Huang et al., 2021). Fan et al. synthesized Lac-BSA nanoparticles loaded with celastrol (CEL), a triterpenoid metabolite from *Tripterygium wilfordii* Hook.f. (Celastraceae). These nanoparticles activated the AMPK/SIRT1 pathway, thereby effectively reducing lipid accumulation, restoring liver function, and improving insulin sensitivity (Fan et al., 2022). Nootkatone (Nok) is a sesquiterpene ketone naturally found in *Alpinia oxyphylla* Miq. (Zingiberaceae) (Wang Y. et al., 2018). Yong et al. demonstrated that Nok markedly ameliorated lipid and glucose metabolic disorders in MAFLD mice and alleviated liver injury. In PO-stimulated L02 cells, Nok upregulated the expression of p-AMPK and p-ACC, whereas the specific inhibitor Compound C reversed the activation of p-AMPK induced by Nok (Yong et al., 2022). Geniposide (GEN) is a plant metabolite isolated from the fruits of *Gardenia jasminoides* J.Ellis (Rubiaceae). Shen et al. found that GEN ameliorated tyloxapol-induced lipid accumulation and inflammatory injury in mice and lowered the overexpression of the inflammatory factors. Western blot analysis revealed that GEN enhanced the phosphorylation of AKT and AMPK, while suppressing the levels of P-mTOR and SREBP-1c. This study revealed that GEN alleviates MAFLD-caused oxidative stress and inflammation, mainly through modulation of the AMPK/PI3K/AKT/mTOR pathway (Meireles et al., 2022; Qian et al., 2024; Shen et al., 2020). Catalpol extracted from *Rehmannia glutinosa* (Gaertn.) Libosch. ex DC (Orobanchaceae) attenuated hepatic steatosis by promoting fatty acid β-oxidation via an AMPK-dependent mechanism (Tian et al., 2020).

### 3.5 Phenols

Gallic acid (GA) derived from *Rheum palmatum L* (Polygonaceae) is effective in ameliorating MAFLD (Chao et al., 2014; Singh and Chauhan, 2013). Zhang et al. demonstrated through clinical specimen analysis and GEO database mining that activation of the AMPK-ACC-PPARα pathway is strongly associated with MAFLD progression. We found that H&E staining showed that GA treatment markedly reduced cytoplasmic fat vesicles and balloon-like degeneration. Oil red O staining further confirmed suppressed lipid droplet accumulation in hepatic tissue. Masson staining revealed alleviated ballooning degeneration in mouse livers, a core histological index for fibrosis assessment. In *vitro* experiments, GA effectively decreased the number of lipid droplets as well as TG and TC levels in OA/PA-stimulated hepatocytes. Western blot assays showed GA upregulated p-AMPK, p-ACC, and PPARα expression. The same results were obtained in *in vivo* experiments. Next, cellular AMPK expression was silenced via siAMPK transfection. Western blot results confirmed that AMPK knockdown significantly reversed the regulatory effect of GA on p-AMPK expression in hepatocytes. Excessive hepatic lipid uptake generates excess oxidative substrates, leading to excessive ROS production. Mitochondrial membrane potential (MMP) in live cells was detected by flow cytometry and laser scanning confocal microscopy. GA treatment efficiently recovered depleted MMP to baseline values, while AMPK knockdown via siAMPK completely eliminated GA’s antioxidant capacity. Collectively, these results illustrate that GA alleviates hepatic lipid deposition and aberrant ROS overproduction via activation of the AMPK-ACC-PPARα pathway (Zhang N. et al., 2023; Zhang J. et al., 2023). Salvianolic acid A (SAA) is a water-soluble phenolic metabolite isolated from the root of *Salvia miltiorrhiza* Bunge (Lamiaceae). Zhu et al. established an MAFLD model using Western diet-fed diabetic ApoE mice, in which SAA treatment inhibited lipid accumulation by modulating the AMPK pathway (Zhu et al., 2024). Teng et al. constructed liver-targeted oxidized starch lysozyme (OSL) nanoparticles, which were used to realize the targeted delivery of resveratrol (Res), a stilbene metabolite from *Reynoutria japonica* Houtt (Polygonaceae), to liver tissue. This study adopted an HFD-induced mouse MAFLD model. The results reveal that Gal-OSL/Res effectively ameliorates hepatic lipid deposition and IR via regulation of the AMPK/SIRT1/FAS/SREBP1c pathway (Teng et al., 2019). Isostrictiniin (ITN) is a phenolic metabolite isolated from *Nymphaea candida* C.Presl (Nymphaeaceae). Accumulating studies have confirmed that ITN ameliorates glycolipid metabolic disorders in the MAFLD model mice via regulation of the AMPK/SREBP-1c/ACC signaling pathway. In addition, ITN suppresses hepatic inflammatory responses and alleviates oxidative stress, ultimately reversing pathological hepatic steatosis (Yan et al., 2024).

### 3.6 Others

Levo-tetrahydropalmatine (L-THP) is one of the major metabolites derived from *Corydalis yanhusuo* (Y.H. Chou and C.C. Hsu) W.T. Wang ex Z.Y. Su and C.Y. Wu (Papaveraceae). Guo et al. established a MAFLD-related injury model in BEL-7402 hepatocytes via photooxidation (PO) induction. L-THP effectively minimized the number of lipid droplets, downregulated TC and TG contents, and alleviated hepatocyte lipotoxicity. Western blot analysis showed that L-THP effectively upregulated the protein levels of SIRT1 and p-AMPK/AMPK, while downregulating FAS and HIF-1α protein expression. Collectively, THP ameliorates hepatic lipotoxicity via activating the AMPK/SIRT1 pathway (Guo et al., 2023). Psoralen is the main metabolite derived from the traditional Chinese medicine *Cullen corylifolium* (L.) Medik. (Fabaceae). Wang et al. used sodium oleate to induce MAFLD in L02 cells. Psoralen can increase the level of p-LKB1, p-AMPK, and p-ACC, and dramatically raise the LC3II/LC3I ratio, while the autophagy inhibitor chloroquine can block psoralen’s improvement of cellular lipid deposition. Compound C was further employed and was found to downregulate p-AMPK. In addition, AMPKα siRNA dramatically decreased AMPK expression and reversed psoralen-induced downregulation of FASN and SREBP1 as well as the increase in LC3II/LC3I ratio. Meanwhile, intracellular lipid accumulation was further aggravated. It was demonstrated that psoralen ameliorates MAFLD through the LKB1/AMPK signaling pathway by promoting autophagosome synthesis and autophagic flux (Wang et al., 2022). Emodin is an anthraquinone-derived metabolite extracted from *Pleuropterus multiflorus* (Thunb.) Turcz. ex Nakai (Polygonaceae). Wang et al. verified that emodin indirectly activates AMPK via CaMKKβ, thereby inhibiting the mTOR pathway and ameliorating hepatic steatosis (Wang et al., 2017). Yu et al. established an MAFLD zebrafish model fed with egg yolk powder. They confirmed that emodin is the primary metabolite in *Pleuropterus multiflorus* (Thunb.) Turcz. ex Nakai for treatment of MAFLD, and it promotes fatty acid oxidation by activating the AMPK pathway (Yu et al., 2020).

In summary, several experimental investigations have demonstrated that many plant metabolites can directly or indirectly activate the AMPK pathway and play a therapeutic role in the treatment of MAFLD. This review aims to offer experimental and theoretical references for clinical therapy and the development of novel traditional Chinese medicines. The mechanisms whereby plant metabolites target the AMPK pathway to intervene in MAFLD are summarized in Table 1.

**TABLE 1:** Plant metabolites regulate the AMPK signaling pathway for the treatment of MAFLD.

Classification	Metabolites	Subjects	Dose	Time	Positive control	Mechanism of action	Pathway	References
Saponins	Ginsenoside Rb2	Mice	10 mg/kg	4 weeks	—	Enhance autophagy and reduce hepatic lipid accumulation	AMPK/mTOR signaling pathway	Huang et al. (2017)
	Ginsenoside Rg1	HepG2 cell	40/80 μg/mL	24 h	—	Reduced lipid accumulation and anti-inflammatory effects	AMPK/NF-κB signaling pathway	Xiao et al. (2019a), Xiao et al. (2019b)
	Ginsenoside Rg5	Mice	50/100 mg/kg	3 months	—	Promotes lipolysis, increases autophagy, improves inflammation	Activates the LKB1/AMPKα/PPARγ pathway, while AMPK negatively regulates the mTOR and NF-κB pathways	Shi (2024)
Flavonoids	Naringenin	*In vivo*: SD rats, in vitro: L02 hepatocytes, 3T3-L1 adipocytes	*In vivo*: 10/30/90 mg/kg; in vitro: L02 hepatocytes:0/0.7/2.2/6.7/20μm; 3T3-L1 adipocytes:0/2.2/6.7/20 μM	*In vivo*: 2 weeks; i*n vitro*: 24 h	*In vivo*: Metformin	Reduce hepatic lipid deposition	CaMKKβ/AMPK signaling pathway, directly bind to the AMPKγ1 subunit	Yang et al. (2021)
	Atractylenolide III	Mice, HepG2 cell, AML12 murine hepatocyte cell line	*In vivo*: 10 mg/kg *In vitro*: 12.5/25/50 μg/mL	*In vivo*:4 weeks; *In vitro*: 24 h	—	Reduces lipid accumulation, Ameliorates oxidative stress damage	AdipoR1/LKB1/AMPK/SIRT1 signaling pathway	Li et al. (2022)
	Scutellarin	Rat, LO2 cells	*In vivo*: 25/50/100 mg/kg *In vitro*: 25/50/100 μM	*In vivo*: 8 weeks; *In vitro*: 24 h	*In vivo*/*In vitro*: Curcumin	Reduces blood glucose levels, decreases lipid aggregation and fibrosis of the cells	SIRT3/AMPK/ACC signaling pathway	Zhou et al. (2024)
	Galangin	*In vivo*: Male rats, in vitro: Human hepatic stellate cells LX-2	*In vivo*: 25/50/100 mg/kg *In vitro*: 25/50/100 μM	*In vivo*: 4 weeks *In vitro*:24 h	—	Improvement of hepatic fat accumulation and fibrosis	AMPK signaling pathway	Cen et al. (2022)
	Baicalein	Rat	25/100 mg/kg	5 weeks	—	Ameliorate lipid accumulation in the liver	AMPK/PGC1α and PPARα signaling pathways	Zhang J et al. (2023)
	Puerarin	Mice	75/150/300 mg/kg	4 weeks	Metformin	Improves hepatic lipid deposition, oxidative stress, and inflammation	AMPK/Nrf2/NF-κB signaling pathway	Han et al. (2026)
Polysaccharides	*Arctium lappa* L. (Asteraceae) polysaccharide	HepG2 cell	5/50/100/300 μg/mL	24 h	—	Reduce lipid droplet accumulation, \anti-inflammatory	AMPK signaling pathway	Zeng et al. (2023)
	*Astragalus mongholicus* Bunge (Fabaceae) polysaccharide	Mice	800 mg/kg	12 weeks	Metformin	Reduce IR and lipid accumulation in the liver	AMPK signaling pathway	Sun et al. (2019)
	Pachymaran	Mice	50/100/200 mg/kg	8 weeks	—	Improve steatosis, lymphocyte and macrophage infiltration	CaMKKβ/AMPK signaling pathway	Yu et al. (2025)
Terpenoids	Paeoniflorin	Rat	10/20/40 mg/kg	8 weeks	Pioglitazone	Enhances insulin sensitivity and promotes β-oxidation Lowers insulin, glucagon, inhibits lipogenesis	LKB1/AMPK signaling pathway	Li et al. (2018)
	Alisol A	Mice	100 mg/kg	8 weeks	Rimonabant	Reduce lipid deposition, enhance glucose tolerance and insulin sensitivity	AMPK/SREBP-1c signaling pathway	Ho et al. (2019)
	Triptolide	Mice	50 μg/kg per day is effective and non-hepatotoxic, while 100 μg/kg per day causes severe hepatotoxicity	10 weeks	—	Improve lipid metabolism disorders in the liver	AMPK/ACC1 signaling pathway	Huang et al. (2021)
	Celastrol	Mice	1 mg/kg	4 weeks	—	Reduce lipid deposition and improve liver function Enhance insulin sensitivity	AMPK/SIRT1 signaling pathway	Fan et al. (2022)
	Nootkatone	Mice, L02 cell	*In vivo*: 25/50 mg/kg/d, in vitro: 5/10/40 μM	*In vivo*: 12 weeks; *in vitro*: 24 h	*In vitro*: Metformin	Improve lipid accumulation	AMPK/ACC signaling pathway	Yong et al. (2022)
	Geniposide	Mice	50/75/100 mg/kg	19 h	Fenofibrate	Improve lipid accumulation and inflammatory damage	AMPK/PI3K/AKT/mTOR signaling pathway	Shen et al. (2020)
	Catalpol	*In vivo*: mice, *in vitro*: HepG2 cell	*In vivo*: 100/200/400 mg/kg, in vitro: 100/200/400 μM	*In vivo*: 18 weeks; *In vitro*: 24 h	Atorvastatin calcium	Enhance the β -oxidation of fatty acids Improve hepatic steatosis	AMPK/ACC signaling pathway	Tian et al. (2020)
Phenols	Gallic acid	*In vivo*: mice, in vitro: HepG2, SMMC-7721 hepatocytes	*In vivo*: 50/100 mg/kg *In vitro*: 20 μM	*In vivo*: 12 weeks *In vitro*: 24 h	—	Reduce lipid accumulation in the liver and ROS	AMPK-ACC-PPARα signaling axis	Zhang J et al. (2023)
	Salvianolic acid A	Diabetic ApoE mice	10/20 mg/kg	Continuous administration during modeling	—	Reduce lipid accumulation	AMPK signaling pathway	Zhu et al. (2024)
	Resveratrol	Mice	20 mg/kg	8 weeks	—	Improve lipid deposition and IR	AMPK/SIRT1/FAS/SREBP1c signaling pathway	Teng et al. (2019)
	Isostrictiniin	Mice	25/50/100 mg/kg	4 weeks	Atorvastatin calcium	Alleviate hepatic lipid deposition, IR, hepatic injury, oxidative stress and inflammatory response	AMPK/SREBP-1c/ACC signaling pathway	Yan et al. (2024)
Others	Levo-tetrahydropalmatine	BEL-7402 human hepatoma cell	75/100 μmol/L	24 h	—	Reduce lipid accumulation	AMPK/SIRT1 signaling pathway	Guo et al. (2023)
	Psoralen	L02 cell	0.37/1.1/3.3 μM	24 h	—	Reduce lipid deposition, Increase autophagy	LKB1/AMPK/ACC pathway	Wang et al. (2022)
	Emodin	Rat, zebrafish	40/80/160 mg/kg; 0.25/0.5 μg/mL	*In vivo*: 8 weeks; 72 h	—	Improve hepatic steatosis, Increase the oxidation of fatty acids	CaMKKβ-AMPK-mTOR signaling pathway, AMPK signaling pathway	Wang et al. (2017), Yu et al. (2020)

## 4 Discussion

MAFLD is currently regarded as the hepatic manifestation of metabolic disorders. Its prevalence is increasing worldwide along with the epidemics of diabetes and obesity (Sangro et al., 2023). Recently, AMPK, an essential regulator in maintaining energy metabolic homeostasis, has demonstrated significant therapeutic prospects in multiple disease fields, including metabolic disorders, cardiovascular and cerebrovascular diseases, and malignancies (Entezari et al., 2022; Wang Q. et al., 2018; Wu and Zou, 2020). At present, several AMPK activators have been approved for marketing or are in clinical trials. Metformin is already the first-line drug prescribed for type II diabetes in clinical practice and has the potential to enter the fields of aging, cancer, and cardiovascular diseases (LaMoia and Shulman, 2021). Studies have revealed that metformin exerts its therapeutic effects by binding to PEN2, thereby activating the AMPK pathway (Ma et al., 2022). However, although metformin remains the drug of choice, it still has limitations in improving liver function, alleviating liver fibrosis, and regulating lipid metabolism in patients with type 2 diabetes complicated by MAFLD (Jiang and Zhao, 2025). MK-8722, a potent direct pan-AMPK activator, has been identified and shown to activate 12 mammalian AMPK complexes. However, its long-term use was associated with cardiac hypertrophy and increased myocardial glycogen accumulation (Myers et al., 2017).

**FIGURE 8:**
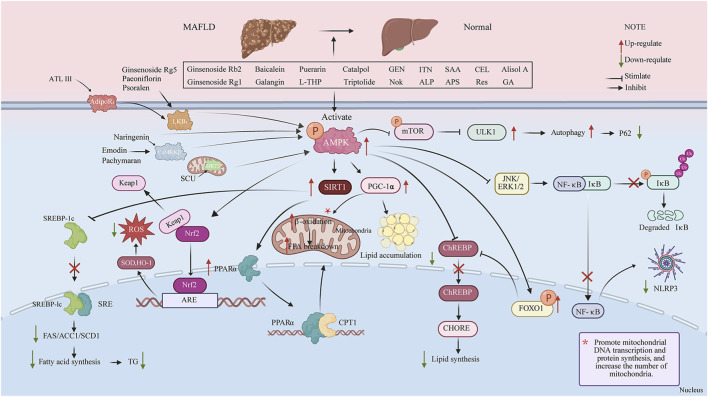
Schematic overview of the regulatory network by which plant metabolites target AMPK to ameliorate MAFLD.

Given the safety and efficacy drawbacks of synthetic AMPK activators, researchers have turned to widely accessible plant metabolites with fewer side effects. In this paper, we systematically described that the modulation of AMPK signaling pathway by plant metabolites is involved in the treatment of MAFLD. This occurs through various pathways such as improving IR, inhibiting adipogenesis, attenuating inflammation, resisting oxidative stress injury, enhancing autophagy, and improving mitochondrial function. Unlike previous studies that primarily focused on superficial phenotypic observations, the present work further clarifies the upstream mechanisms, direct/indirect activation patterns, and functional specificity of AMPK modulation by plant metabolites. Specifically, paeoniflorin indirectly activates the AMPK signaling pathway by upregulating the upstream key kinase LKB1. It subsequently promotes fatty acid β-oxidation and inhibits hepatic lipogenesis, thereby alleviating hepatic lipid accumulation in MAFLD. In contrast, naringenin exerts dual regulatory effects on AMPK. It indirectly activates AMPK via the upstream CaMKKβ-mediated signaling cascade, and also directly binds to the AMPKγ1 subunit, leading to specific AMPK pathway activation. This represents a distinct targeted regulatory feature compared with paeoniflorin. Overall, this study elucidates the unique molecular mechanisms by which different plant metabolites modulate MAFLD via AMPK targeting, avoiding the simplistic and homogeneous phenotypic descriptions commonly reported in previous literature. These findings demonstrate that the AMPK pathway represents a highly promising therapeutic target for MAFLD, thereby offering a scientific basis for the discovery of targeted traditional Chinese medicine for MAFLD. To intuitively summarize the abovementioned AMPK-centered molecular regulatory network in MAFLD and clarify the distinct target characteristics of plant metabolites including paeoniflorin and naringenin, an integrated schematic diagram (Figure 8) was included in this study.

Numerous studies have demonstrated that various plant metabolites exert regulatory effects on the AMPK signaling pathway. However, current research has notable limitations. Although a small number of studies perform reverse functional validation using AMPK siRNA knockdown and Compound C inhibitor, most publications only measure p-AMPK levels via Western blot. Mechanistic conclusions are drawn solely from correlative expression changes, without complete evidence chains to confirm causal relationships. This flaw undermines the reliability of the AMPK-centered regulatory network summarized in this review. Meanwhile, *in vitro* experiments predominantly utilize immortalized HepG2 and L02 cell lines, lacking validation data from primary hepatocytes. Such oversimplified experimental models markedly reduce the translational reference value of experimental outcomes for clinical MAFLD treatment. These methodological limitations are further aggravated by the fact that positive control groups are omitted in the majority of included studies. Without a unified benchmark for pharmacodynamic comparison, researchers cannot quantify the therapeutic potency of distinct plant metabolites. Neither can they establish internal quality control standards for experimental systems. Moreover, most studies focus on upstream or downstream targets of the pathway, while failing to fully consider the interactions among different targets, and thus cannot thoroughly elucidate its multi-layered and complex molecular regulatory network. Furthermore, current preclinical investigations of plant metabolites are largely restricted to conventional *in vitro* cellular and *in vivo* animal models. Comprehensive assessments regarding pharmacokinetic profiles, oral bioavailability, *in vivo* metabolic stability, systemic toxicity, tissue-targeting property and long-term safety remain insufficient for these metabolites. The scarcity of relevant clinical trials further hampers the verification of their therapeutic efficacy and safety against MAFLD, substantially restricting their prospects for clinical translation. In addition, several practical issues still need to be addressed urgently, including the standardization of raw medicinal materials of Chinese herbs, rational conversion between animal experimental doses and human equivalent doses, and the establishment of clinical application systems. Collectively, these translational deficiencies have become major obstacles restricting the clinical popularization and new drug development of AMPK-targeted plant metabolites for MAFLD treatment.

To address the above deficiencies in translational research, future investigations should first remedy prominent methodological flaws in existing experimental designs. First, researchers should construct complete causal evidence chains by combining correlative protein detection with loss-of-function experiments such as AMPK gene silencing and pharmacological inhibition, rather than drawing mechanistic conclusions merely based on changes in p-AMPK expression. Second, primary hepatocytes should be used alongside immortalized cell lines for cross-verification to improve the clinical similarity of experimental models. Third, standardized positive control groups must be set up to provide unified pharmacodynamic benchmarks for quantitative comparison of the efficacy of different plant metabolites and stabilize experimental quality control. With solid experimental quality guaranteed, future research can further adopt innovative and holistic strategies to resolve deeper translational bottlenecks. Beyond standardized experimental design, research should shift from fragmented pathway analysis toward holistic research strategies based on systems pharmacology and network biology. Specifically, integration of multi-omics technologies including proteomics, metabolomics, and single-cell transcriptomics with advanced bioinformatics enables a more comprehensive elucidation of crosstalk and hierarchical regulatory mechanisms among AMPK-related subnetworks. This approach moves beyond the conventional linear mode focused merely on individual upstream or downstream targets. Regarding experimental systems, the development of patient-derived organoids and humanized animal models bridges the gap between basic research and clinical practice, while reducing reliance on conventional cell and animal models. Clinically, rigorously designed randomized controlled trials are urgently required for standardized herbal metabolites and extracts to verify their clinical efficacy and safety. Meanwhile, standardized pharmacokinetic and toxicokinetic assessments help optimize the oral bioavailability of metabolites. In addition, unified quality control criteria for herbal raw materials, human-animal dose conversion protocols based on body surface area or allometric scaling, and comprehensive clinical supervision systems should be established. From a translational perspective, nanoparticle delivery systems and prodrug modification strategies can enhance the *in vivo* bioavailability of plant metabolites. On this basis, in addition to ongoing investigations into pharmacological mechanisms, future research should prioritize systematic structure-activity relationship (SAR) analysis and rational structural derivatization of natural parent skeletons. Molecular modification strategies can be leveraged to address the inherent limitations of prototype metabolites, including low bioavailability and inadequate target agonistic activity. Taken together, establishing an integrated research framework combining multi-target network regulation, advanced preclinical models, and standardized clinical validation is essential to fully realize the therapeutic potential of plant metabolites targeting AMPK in MAFLD. In conclusion, although natural AMPK modulators show promising prospects, overcoming current research limitations requires holistic, multidisciplinary, and clinically oriented research designs. Such approaches will greatly accelerate the clinical translation and new drug development of plant metabolites for MAFLD prevention and treatment.

## Glossary

MAFLD: metabolic dysfunction-associated fatty liver disease
NAFLD: non-alcoholic fatty liver disease
NASH: non-alcoholic steatohepatitis
IR: insulin resistance
AMPK: AMP-activated protein kinase
DNL: *de novo* lipogenesis
FAO: fatty acid oxidation
PPAR: peroxisome proliferator-activated receptor
TC: total cholesterol
TG: triglycerides
ACC: acetyl-CoA carboxylase
LKB1: liver kinase B1
CPT1: carnitine palmitoyltransferase 1
mTOR: mammalian target of rapamycin
SIRT1: sirtuin 1
PPARα: peroxisome proliferator-activated receptor alpha
ROS: reactive oxygen species
Nrf2: nucleus factor erythroid 2-related factor 2
ER: endoplasmic reticulum
PI3K: phosphatidylinositol 3-kinase
AKT: protein kinase B
CaMKKβ: calmodulin-dependent protein kinase β
IRS-1: insulin receptor substrate-1
NQO1: NAD(P)H:quinone oxidoreductase 1
SOD: superoxide dismutase
GSH-Px: glutathione peroxidase
TXNIP: thioredoxin-interacting protein
FOXO3: forkhead box protein o3
ULK1: unc-51 like autophagy activating kinase 1
TSC2: tuberous sclerosis 2
TET2: tet methylcytosine dioxygenase 2
ATL III: Atractylenolide III
SCU: Scutellarin
ALP: *Arctium lappa* L. (Asteraceae) polysaccharide
APS: *Astragalus mongholicus* Bunge (Fabaceae) polysaccharide
CEL: celastrol
Nok: Nootkatone
GEN: Geniposide
GA: Gallic acid
SAA: Salvianolic acid A
NLRP3: nod-like receptor family pyrin domain containing 3
IL-1β: interleukin 1 beta
IL-18: interleukin 18
JNK: c-jun n-terminal kinase
Erk1/2: extracellular signal-regulated kinase 1/2
TNF-α: tumor necrosis factor alpha
IL-6: interleukin 6
ATP: adenosine triphosphate
KEAP1: kelch-like ech-associated protein 1
ARE: antioxidant response element
HO-1: heme oxygenase 1
L-THP: Levo-tetrahydropalmatine
ITN: Isostrictiniin
Res: resveratrol
